# Biohybrid cochlear implants in human neurosensory restoration

**DOI:** 10.1186/s13287-016-0408-y

**Published:** 2016-10-07

**Authors:** Ariane Roemer, Ulrike Köhl, Omid Majdani, Stephan Klöß, Christine Falk, Sabine Haumann, Thomas Lenarz, Andrej Kral, Athanasia Warnecke

**Affiliations:** 1 Department of Otorhinolaryngology, Head and Neck Surgery, Hannover Medical School, Carl-Neuberg-Str. 1, 30625 Hannover, Germany; 2 Institute for Cellular Therapeutics, IFB-Tx, Hannover Medical School, Carl-Neuberg-Str. 1, 30625 Hannover, Germany; 3 Institute of Transplant Immunology, IFB-Tx, Hannover Medical School, Carl-Neuberg-Str. 1, 30625 Hannover, Germany; 4 Cluster of Excellence “Hearing4all”, Carl-Neuberg-Str. 1, 30625 Hannover, Germany

**Keywords:** Cochlear implants, Biohybrid electrode, Hearing loss, Bone marrow-derived mononuclear cells

## Abstract

**Background:**

The success of cochlear implantation may be further improved by minimizing implantation trauma. The physical trauma of implantation and subsequent immunological sequelae can affect residual hearing and the viability of the spiral ganglion. An ideal electrode should therefore decrease post-implantation trauma and provide support to the residual spiral ganglion population. Combining a flexible electrode with cells producing and releasing protective factors could present a potential means to achieve this. Mononuclear cells obtained from bone marrow (BM-MNC) consist of mesenchymal and hematopoietic progenitor cells. They possess the innate capacity to induce repair of traumatized tissue and to modulate immunological reactions.

**Methods:**

Human bone marrow was obtained from the patients that received treatment with biohybrid electrodes. Autologous mononuclear cells were isolated from bone marrow (BM-MNC) by centrifugation using the Regenlab™ THT-centrifugation tubes. Isolated BM-MNC were characterised using flow cytometry. In addition, the release of cytokines was analysed and their biological effect tested on spiral ganglion neurons isolated from neonatal rats. Fibrin adhesive (Tisseal™) was used for the coating of silicone-based cochlear implant electrode arrays for human use in order to generate biohybrid electrodes. Toxicity of the fibrin adhesive and influence on insertion, as well on the cell coating, was investigated. Furthermore, biohybrid electrodes were implanted in three patients.

**Results:**

Human BM-MNC release cytokines, chemokines, and growth factors that exert anti-inflammatory and neuroprotective effects. Using fibrin adhesive as a carrier for BM-MNC, a simple and effective cell coating procedure for cochlear implant electrodes was developed that can be utilised on-site in the operating room for the generation of biohybrid electrodes for intracochlear cell-based drug delivery. A safety study demonstrated the feasibility of autologous progenitor cell transplantation in humans as an adjuvant to cochlear implantation for neurosensory restoration.

**Conclusion:**

This is the first report of the use of autologous cell transplantation to the human inner ear. Due to the simplicity of this procedure, we hope to initiate its widespread utilization in various fields.

## Background

According to the World Health Organization, about 5 % of the world’s population, i.e., more than 300 million people, suffer from disabling hearing loss. For rehabilitation of severe to profound hearing loss, patients are treated with cochlear implantation. Currently, four manufacturers (MedEl, Advanced Bionics Cooperation, Cochlear Cooperation and MXM/Neurolec/Oticon) are FDA approved and provide different electrode designs [1, 2]. In addition, novel companies providing cochlear implants, e.g., the Venus cochlear implant system provided by Nurotron Biotechnology, are emerging. All available implants share common key features: an external device for conversion of acoustic energy into an electrical signal [1–4]. This signal is transmitted subcutaneously to an implanted internal signal receiver that is used to drive the stimulation of the spiral ganglion neurons [3]. An electrode carrier consisting of platinum contacts embedded in silicone is connected to this implanted device and is inserted into the scala tympani of the cochlea [3]. Differences between the devices include the length, the stiffness, and the design of the electrode. For electrical stimulation of the auditory system, a full circuit loop from an active electrode to a second return electrode is necessary [1]. The mode of activation is either monopolar or bipolar [3]. An inactive reference electrode located outside the cochlea is needed for monopolar stimulation, whereas bipolar stimulation is performed by using two neighbouring intracochlear electrodes [3]. A recent survey has evaluated the complications after cochlear implantation showing that the overall incidence is about 20 % [5]. Infection of the skin flap covering the implant, seroma or hematoma, foreign body reaction, tinnitus, disequilibrium, device failure, neurological complications such as facial palsy or dysgeusia, cholesteatoma, migration of the device, recurrent otitis media, and chronic headaches are among the reported complications [5–7]. A systematic review of the literature showed that severe complications related to infection leading to mastoiditis and meningitis are very rare when performed by an experienced surgeon [6]. The incidence of disequilibrium is higher in elderly patients aged 75 years or above [8]. Vestibular complications show the highest incidence when regarding delayed complications after cochlear implantation [6]. Overall, cochlear implantation is considered as a safe surgical technique for hearing restoration [5].

Speech understanding among cochlear implant listeners is highly variable and rarely predictable. The quality of the interface between the electrodes and the spiral ganglion neurons is one of the main factors affecting speech understanding [9]. The position of the electrode array within the cochlea and the trauma resulting during electrode insertion strongly influence the nerve-electrode interaction and therefore can affect speech perception [9–13].

Despite significant advancements in electrode design and surgical implantation techniques, insertion trauma still has the potential to negatively impact hearing outcomes. Direct tissue damage as well as immunological reactions after implantation lead to fibrotic and osteogenic [14] alterations of the scala tympani. The amount of fibrous and osseous tissue is negatively correlated with residual spiral ganglion neuron counts in humans [10]. Temporal bone pathology studies have shown that outcome measures such as word recognition scores depend on the number of residual spiral ganglion neurons [10, 12]. Thus, insertion trauma may affect the outcome of sensory restoration [15] and require higher electrical stimuli for effective neurostimulation [13]. Other negative outcomes of trauma include increased energy consumption and aberrant spread of current resulting in cross-channel interactions [9, 13]. Atrophy of the stria vascularis arising from tissue damage or ageing also can impair implant performance since it influences the health of the organ of Corti and consequently also the state of the neurons to be excited by the cochlear implant [16].

Cells derived from the bone marrow possess the innate capacity to induce repair of traumatised tissue and to modulate immunological reactions [17–19]. The microenvironment of bone marrow includes a mixed population of stromal cell types that are possible sources of chemokines, growth factors, and cytokines [20]. Diverse studies have evaluated the use of mesenchymal progenitor or embryonic stem cells [18, 19, 21, 22] and only a few considered hematopoietic progenitor cells for the restoration of the inner ear [22, 23]. However, none of these cells have yet been used in the clinical setting for inner ear therapy. Hematopoietic progenitor cells are able to migrate into the cochlea even after intravenous transplantation of whole bone marrow [22]. This effect has not been observed for mesenchymal progenitor cells (MPC). MPC secrete a variety of neuroprotective growth factors [17] and reduce scarring by downregulation of excessive fibroblast proliferation [24]. The secretome of progenitor cells isolated from the bone marrow can stabilise traumatised tissue [17]. Injuries occur during electrode insertion and are responsible for ossification of the cochlea [14]. Therefore, anti-inflammatory treatment may be crucial for hearing preservation and for the prevention of fibrotic tissue formation [25].

Potentially, this anti-inflammatory treatment could be achieved by delivering autologous progenitor cells along with a cochlear implant. The aim of the present study was to develop a clinically feasible protocol for the generation of a biohybrid electrode using autologous mononuclear cells derived from the bone marrow (BM-MNC). The term BM-MNC includes all cells with unilobulated or rounded nuclei that lack granules in the cytoplasm [26]. Due to their density and size, BM-MNC can be easily separated from myeloid cells and erythrocyte progenitors [26]. They consist of hematopoietic progenitor cells at different stages of maturation [27]. Other cells contributing to the BM-MNC are cells with multipotent capacity such as mesenchymal stromal cells [20, 27], very small embryonic-like stem cells [28, 29], multipotent adult progenitor cells [30], endothelial progenitor cells [31], and tissue-committed stem cells [27]. In addition to the progenitor cells, lymphocytes, plasmatic cells, monocytes, and macrophages also reside in the bone marrow and can be identified in the mononuclear fraction [26]. Due to their capacity to exert neuroprotection, immunomodulation, neurorestoration, and neurogenesis, autologous BM-MNC were transplanted into patients with cerebral palsy and led to a reduction in disability and to an improvement in the quality of life [32]. In addition, a short-term benefit of infusing bone marrow-derived cells into patients with chronic heart failure has been observed, and this effect seems to be related to the secretome of these cells [33]. Thus, BM-MNC have been widely used in human studies as an immune modulator and source of protective growth factors. The goal of the this study was to demonstrate the feasibility and safety of this approach in human cochlear implantation. As part of this study, autologous BM-MNC were attached to cochlear implant electrodes using fibrin glue. For each patient, safety readouts consisted of regular physical examination, impedance measures of the electrode in the implanted ear, and comparison to the contralateral, previously implanted ear. An aliquot of BM-MNC from each patient was used to study persistence of cells on a coated electrode in vitro and to assay their protective qualities.

## Methods

Human BM-MNC were used for all experiments and procedures and were isolated from the cochlear implant recipients described below. Ethical approval from the ethics committee of the Hannover Medical School was obtained, as well as written informed consent from all patients.

### Isolation of BM-MNC

In order to obtain bone marrow from cochlear implant patients, a sternal puncture was performed under general anaesthesia just prior to insertion of the cochlear implant. All instruments which were in contact with the bone marrow were washed with heparin solution (100 U/ml). About 8–10 ml bone marrow was obtained through aspiration and 8 ml was transferred into a RegenKit®-THT (Regen Lab, Lausanne, Switzerland) tube. The tube was centrifuged in the Regenlab Centrilab® (Regen Lab) at 3300 rounds per minute for 10 mins. By inverting the tube gently three to four times, the mononuclear cell fraction was re-suspended in the supernatant. This cell-containing supernatant is referred to as a mononuclear cell suspension.

One aliquot was used for the generation of the biohybrid electrode and subsequent implantation. Another aliquot of the supernatant prior to re-suspension of the mononuclear cell fraction as well as the remaining cell suspension were collected for the in vitro experiments described below.

For experimental in vitro studies, the cells were cultured in a medium consisting of 78 ml DMEM (Biochrome GmbH, Berlin, Germany), 1 ml penicillin (30 U/ml, Biochrome GmbH), 20 ml fetal calf serum (FCS; Hyclone, Thermoscientific, Waltham, Massachusetts) and 1 ml Hepes-buffer solution (Invitrogen, Germany). This medium is referred as to MNC medium.

### Experimental in vitro studies

#### Analysis of the BM-MNC

For cellular analysis of the components of the BM-MNC of the first two patients, a novel flow cytometric assay was used to characterise different cell fractions on a 10-colour flow cytometer (Navios, Beckman Coulter). Cell counting was carried out with an automated Hemocounter (Coulter ACTdiff, Beckman Coulter). The cells were analysed for viability by flow cytometry (7-aminoactinomycin D (7-AAD); Beckman Coulter) to distinguish viable from dead/apoptotic cells whereas cell debris were discriminated by cell scatter properties (forward and side scatter) and setup adjustments via the cell discriminator integrated in the Beckman Coulter software (Navios™Cytometry List Mode Data Acquisition&Analysis software). In vitro diagnostic (IVD)- and/or analyte-specific reagent (ASR)-fluorescence-conjugated monoclonal antibodies (mABs), especially CD166, CD105, CD73, and CD90, were used as known markers for putative mature mesenchymal stem cells after depletion. CD45, CD3, CD34, and CD14 were utilised to discriminate the negative from the positive cell phenotypes of the different leukocyte (CD45^+^) and non-leukocyte (CD45^–^) cell subpopulations in the cytometric measurements. All mABs were purchased from Beckman Coulter and Pharmigen™ and are listed in Table 1. After staining with these multiple mABs (15 mins, room temperature), the labelled cell samples were treated with lysing solution (15 min, IOTest 3, room temperature; Beckman Coulter) to eliminate red blood cells; 5000 to 10,000 events were acquired after discrimination of debris for 7-AAD^–^, CD45^+^ and accordingly CD45^–^ cell regions.

**Table 1:** Content of mesenchymal and hematopoietic stem cells in human bone marrow-derived mononuclear cell fraction

Conjugated antibody	Catalog number/Company	IVD/ASR/RUO	Fluorochrome	Clone
CD3-ECD	A07748/Beckman Coulter	IVD	ECD	UCHT1
CD14-PB	PN B00846/Beckman Coulter	ASR	PB	RMO52
CD34-PE	A07776/Beckman Coulter	IVD	PE	581
CD45-KO	IM3548/Beckman Coulter	IVD	PC7	J33
CD73-FITC	561254/BD Pharmingen™	RUO	FITC	AD2
CD90-FITC	PN IM1839U/Beckman Coulter	ASR	FITC	F15-42-1-5
CD105-APC	562408/BD Pharming™	RUO	APC	266
CD166-PE	PN A22361/Beckman Coulter	ASR	PE	3A6
HLA-DR-PB	PN A74781/Beckman Coulter	ASR	PB	Immu-357

Process performance was estimated by evaluations of the CD45^–^ and CD45^+^ cell recovery (%), and viability (%) of collected samples from two runs after bone marrow depletion. Individual values are represented from both processes including the percentage of total CD45^+^ and CD45^–^ cells, and the characterisations of viable progenitor cells with positive and negative discrimination markers among viable CD45^–^ cells. *RUO* research use only, *IVD* in vitro diagnostic, *ASR* analyte specific reagent, *ECD* electron coupled dye, *PB* pacific blue, *PE* phycoerythrin, *PC7* phycoerythrin cyanin 7, *FITC* fluorescein isothiocyanate, *APC* allophycocyanin

#### Proteomics studies

For identification of chemokines, cytokines, and growth factors, 200 μl supernatant was obtained after 24 h of cultivation of the suspension of BM-MNC in MNC medium and frozen immediately until analysis. Cytokine levels were measured using the BioRad Bio-Plex Human Angiogenesis Assay (Bio-Rad Laboratories, Inc., Hercules, USA) and Luminex two-laser array reader (Bioplex200, Bio-Rad Laboratories, Inc.). Bioplex Manager 6.1 (Bio-Rad Laboratories, Inc.) was used to acquire standard curves and concentrations.

#### Co-cultivation with spiral ganglion neurons

Spiral ganglion neurons (SGN) were dissected from neonatal Sprague-Dawley rats of both sexes (postnatal days 3–5). After decapitation and removal of the scalp, the skull base was bisected. Under microscopic view, the membranous cochlea was removed and ganglia were collected in an Eppendorf vial filled with Hepes buffer. After centrifugation, Hank’s balanced buffered solution (HBSS; Gibco Invitrogen, Germany) was replaced with 0.01 % trypsin (Biochrom GmbH, Germany) and 0.01 % DNase I (Roche, Germany) in HBSS for the enzymatic dissociation of 30–40 ganglia/2 ml. The solution was incubated at 37 °C for 15 min with intermediate shacking and the cells centrifuged by short-spin followed by the addition of 200 μl FCS (Invitrogen, Germany) in order to stop enzymatic dissociation. The supernatant was removed and the pellet washed three times and re-suspended with serum-free culture medium. Cell yield was defined by counting the cell number in a Neubauer chamber (Brand GmbH, Germany) using trypan blue (Sigma Aldrich, Germany).

The serum-free SGN culture medium consisted of Panserin 401 (Pan Biotech) supplemented with penicillin (30 U/ml; Biochrome GmbH, Germany), phosphate-buffered saline (PBS), 1 M Hepes-buffer (Invitrogen, Germany), glucose (40 %/ml; B. Braun, Germany), insulin (4 mg/ml; Biochrome, Germany), and N2-supplement (Invitrogen, Germany).

In each well of a 48-well-plate, 100 μl spiral ganglion cell solution (containing 0.2 × 10^5^ cells/100 μl) were seeded. Wells were primed with 100 μl supernatant (i.e., the plasma supernatant obtained before re-suspending the MNC by shaking), with 100 μl BM-MNC dissolved in supernatant (BM-MNC), with 100 μl supernatant (i.e., conditioned medium) obtained from BM-MNC cultures after 24 h (cond. med. 24) and with 100 μl supernatant obtained from BM-MNC cultures after 48 h (cond. med. 48). The positive control contained SGN medium supplemented with brain-derived neurotrophic factor (BDNF; 50 ng/ml) and the negative control contained only serum-free culture medium. Each condition was tested in triplets and three independent experiments were performed (*n* = 9).

#### Cytotoxicity tests of fibrin glue

To exclude cytotoxicity of the fibrin adhesive, we tested the fibrin glue composition on spiral ganglion cells (SGC) and MPC. In order to obtain MPC via plastic adhesion from the bone marrow, human BM-MNC were seeded immediately after isolation from the bone marrow (100 μl of the suspension obtained after shaking of the THT tubes) in 48-well plates. After changing the medium, the resulting cell culture consisted only of the cells that attached to the culture well plate via plastic adhesion. These cells were used for the cytotoxicity experiments.

For the cytotoxicity assay, cells (SGC and MPC) were either cultured as a pure cell suspension or mixed with the fibrinogen component of the two-component fibrin adhesive (Tisseal, Illinois, USA) as well as with both components.

The MPC were incubated for 5 days under daily microscopic control. The SGC were cultivated for 2 days. The SGC were fixed after termination with acetone/methanol (AppliChem/Merck, Darmstadt, Germany) 1:1 and labelled with anti-neurofilament antibody for the identification of the neurons. Visualisation was performed after staining with Vectastain ABC kit (Vector) as described in the manual.

The MPC were trypsinised with 0.025 % trypsin-EDTA solution and stained with trypan blue (Biochrome, Germany) 10 %. Quantification of the cells was performed with a Fuchs-Rosenthal counting chamber.

#### Generation of biohybrid (cell-coated) electrodes for in vitro analysis

Nucleus contour advance practice electrodes (Cochlear, Sydney, Australia) were used for these experiments. This electrode consists of 22 half-banded platinum electrode contacts embedded in silicone. An aliquot of 0.5 ml of the BM-MNC suspension was mixed with 0.5 ml of the fibrinogen component of the two-component fibrin adhesive (Tisseal, Illinois, USA). The cochlear implant electrode was pulled through this solution and thereafter through the thrombin component of the two-component fibrin adhesive in order to cover the electrode with a thin layer of cells trapped within.

These model electrodes were used for subsequent experiments. Insertion experiments using a human cochlear model were performed. In addition, coated electrodes were cultivated in MNC medium and microscopic controls were performed regularly up to 4 weeks in order to evaluate the coating success and the biocompatibility of the fibrin adhesive.

#### Measurement of insertion forces during electrode implantation in a three-dimensional cochlear model

Five Nucleus contour advance practice electrodes (Cochlear, Sydney, Australia) were used in a special insertion force-measurement apparatus (designed by Cochlear, Sydney, Australia), a synthetic (polytetraflourethylene) three-dimensional model of the human scala tympani [34]. This model has a depth of 1.5 mm and was constructed using inner and outer wall measurements from silastic casts of human scalae tympani as described by Todd and Naghdy [35]. For each measurement, the electrode was adapted in the special insertion tool and pre-insertion was performed for the first 7 mm. This point was referred to as the starting insertion point and was set at 0 mm. The artificial cochlea was filled with PBS and calibrated. As the electrode was further inserted to its full length, the forces on the outer cochlear wall were measured by INSTRON 5542 (Instron, Massachusetts, USA). Two electrodes were inserted and measured without any coating, whereas three electrodes were coated with BM-MNC as described above. Each electrode was repeatedly inserted and measured five times.

#### Microscopic evaluation of changes in cell morphology due to the coating

In order to assess the proliferation of cells on the electrode surface, MPC were stained with 5(6)-carboxyfluorescein N-hydroxysuccinimidylester (CFSE; Abcam, Cambridge, UK) according to the protocol provided by the manufacturer. Briefly, cells were cultured at standard cell conditions (37 °C, 5 % CO_2_) until they reached confluence (cell number approximately 1 × 10^6^). Cells were then trypsinised and re-suspended several times in fresh culture medium. Thereafter, cells were centrifuged at 800 rpm for 4 min and the supernatant was discarded. Cells were re-suspended with 1 ml PBS containing 10 μM CFSE and were allowed to rest in the solution for 10 min at room temperature. To stop the staining procedure, 1 ml of serum containing MNC medium was added to the cell suspension and pipetted several times prior to centrifugation for the removal of unbound CFSE. Finally, cells were re-suspended with culture medium and the success of the CFSE staining was evaluated using a fluorescence microscope (Olympus, Shinjuku, Japan).

Coating of the electrodes was performed as described above using the stained cells and cultivated for 10 days. Fluorescence microscopy was applied on the 3rd, 7th, and 10th day after coating. Therefore, the medium was replaced with pre-heated PBS and the dye was excited with a light and was detected using the standard FITC filter of the microscope.

### Protocol for human application of biohybrid electrodes

#### Subjects

Three severely hearing-impaired individuals (male; age range 21–43 years) were recruited to receive autologous progenitor cells during their cochlear implantation as salvage treatment. All patients had received a cochlear implant in the contralateral ear with limited success. The audiologic evaluation was performed using our clinical standard procedure as described below. Inclusion criteria were: 1) age 21 years or older; and 2) medical indication for the treatment with a second cochlear implant with expected limited outcome. Exclusion criteria were: 1) ossification of the cochlea after meningitis; and 2) history of malignancy. The choice of the electrode was determined by the contralateral implant type that was used for the first implantation.

#### Patient 1

Patient number 1 (male, 43 years old at the time of the implantation of the biohybrid electrode) suffered from progressive deterioration of hearing after being exposed to hypoxia during birth. The first implanted side showed intelligibility for monosyllabic words measured in silence of 30 % at 65 dB. The left side was considered for implantation with the biohybrid electrode since this side was more severely affected in terms of degree and duration of deafness.

The first side (right side) was provided in 2013 with a Concerto implant with Standard electrode and an Opus2 processor manufactured by MED-EL (Med-EL Elektromedizinische Geräte GmbH, Innsbruck, Austria). The second side (left ear, biohybrid) was implanted with a Synchrony implant with Standard electrode and a Sonnet processor also manufactured by MED-EL.

#### Patient 2

With a long history of progressive hearing loss, patient number 2 received a cochlear implantation as well as revision surgery on the left side (Pulsar implant with Tempo+/later Opus2 processor, CIS strategy, MED-EL GmbH and Nucleus CI24RE(CA) implant with Freedom processor, ACE strategy, Cochlear Ltd.). In 2010, he also received a cochlear implant (Nucleus CI512 implant with CP810 processor, Cochlear Ltd.) on the right side. He presented to our clinic for a second re-implantation on the left side (Nucleus CI512 Profile implant with CP910 processor, Cochlear Ltd., also using ACE strategy) due to insufficient speech understanding. The treatment with BM-MNC was therefor considered for this patient. At the time of the last implantation with the biohybrid electrode, he was 43 years old.

#### Patient 3

With hearing impaired from early childhood, patient number 3 received a cochlear implant on the right side in 2014 at the age of 19 (Nucleus CI24RE (CA) with CP910 processor and MP3000 strategy, Cochlear Ltd.). He wished an implantation on the contralateral side at the age of 21. There is no documentation available about the duration of deafness at the left side and it is thus not known whether this was a pre-, peri- or postlingual deafness. In preoperative tests, he reported some sensation after electrical stimulation of the left cochlear nerve. However, it was not specified whether this was an auditory sensation. Due to the poor performance with the first implant we offered him the implantation in combination with BM-MNC treatment. Thus, he was provided with a Nucleus CI512 Profile implant and a CP910 processor using MP3000 strategy.

#### Surgical procedure

All cochlear implantations were performed in our institution by a single surgeon according to international cochlear implant standards. The standard surgical procedure involves a retroauricular approach, mastoidectomy, posterior tympanotomy, and cochleostomy.

After completion of the cochleostomy, the sternal puncture was performed and the BM-MNC were isolated directly in the operating theatre as described above (see Isolation of BM-MNC). An aliquot of 0.5 ml of the progenitor cell suspension was mixed with 0.5 ml of the fibrinogen component of the two-component fibrin adhesive (Tisseal, Illinois, USA). The cochlear implant electrode was pulled through this solution and thereafter through the thrombin component of the two-component fibrin adhesive in order to cover the electrode with a thin layer of cells trapped within. This biohybrid electrode was inserted immediately in a standard procedure.

To ensure correct electrode function, the electrodes were tested electro-physiologically before insertion, after insertion, and on the second postoperative day.

The electrode position was controlled after insertion via cone beam computed tomography.

#### Audiological evaluation

The evaluation prior to cochlear implantation is standardized and includes pure tone audiogram, speech audiometry with and without the patient’s own hearing aid, acoustic immittance audiometry, otoacoustic emissions, brainstem evoked response audiometry, electrocochleography, and promontory stimulation testing. Also, the function of the vestibular system was evaluated by the caloric test and posturography (EquiTest). All patients underwent preoperative MRI and CT scanning.

During cochlear implant surgery, an impedance measurement was performed. The impedances of the implant electrodes were determined by telemetric measurements according to manufacturer specifications.

Freiburg Speech (polysyllabic numbers and monosyllabic words) at 65 dB as well as a sentence test under quiet and noise conditions (Hochmair-Schulz-Moser; HSM) were used for the assessment of speech discrimination. Measurements were performed 5 weeks and 5 months after implantation. The patients will be evaluated again after 8 months and thereafter annually.

#### Data analysis

Statistical analysis was performed with ORI-GIN 8.0 (OriginLabs, Massachusetts, USA) and GraphPad PRISM (GraphPad Software Inc., California, USA). Repeated measures ANOVA was used with the Bonferroni post hoc test for the correction of *p* values. Data are presented as median or mean with standard deviation.

#### Ethics

The therapeutic protocol for autologous BM-MNC transplantation was presented to and approved by the Institutional Review Board of Hannover Medical School. All performances were done in accordance with the ethical principles for medical research in humans (Declaration of Helsinki). All participants gave a written informed consent after in-depth consultation concerning the possible risks and potential complications of the procedure (e.g., tumour induction, meningitis, and ossification of the cochlea).

Animal studies were approved by the Institutional Animal Care and Research Advisory Committee and by the local state authorities. The study was conducted in accordance with the German ‘Law on Protecting Animals’ and with the European Communities Council Directive 86/609/EEC for the protection of animals used for experimental purposes.

## Results

### Characterisation of BM-MNC based on flow cytometry

**Fig. 1:**
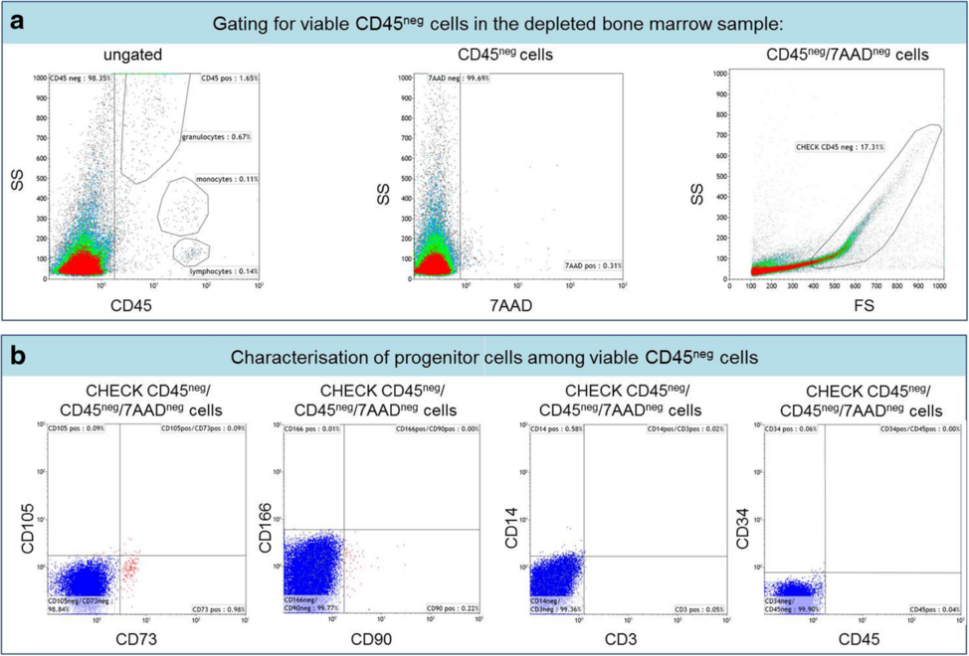
Gating strategy of depleted bone marrow samples for the detection of mesenchymal stem cells. **a** Flow cytometry-based characterisation of viable CD45^–^ cells were discriminated from CD45^+^ leukocytes in the depleted bone marrow (*left histogram*). The *middle histogram* is defined as a region to exclude the 7-aminoactinomycin D (*7AAD*)^+^ dead/apoptotic cells from 7AAD^–^ viable cells. The 7AAD^+^ cells are not further presented in the *right dot plot*, which displays an overview of all scatter event properties and shows differentiations (see *CHECK CD45* ^*neg*^) to non-specifically stained debris by low side (*SS*) and forward scatter (*FS*) signals. **b** In order to detect putative progenitor cells among viable CD45^–^ cells, singularized cells were stained with CD73, CD105, CD166, and CD90 (positive discrimination markers) and CD3, CD14, and CD34 (negative discrimination markers)

Flow cytometry-based characterization of the BM-MNC suspension showed a substantial amount of hematopoietic progenitor cells mixed with mesenchymal and epithelial progenitor cells usable for human application (Fig. 1).

Enrichment of the portion of MPC was achieved using commercially available sterile centrifugation tubes (Regen-Tube, Regenlab, Lausanne, Switzerland). This resulted in a 1.4 log (24.9-fold) augmentation of CD45^–^ cells and a 2.9 log (–827.7-fold) depletion of the CD45^+^ leukocyte cell fraction (Table 2). Human BM-MNC were used for further in vitro analysis as well as for transplantation into the human cochlea.

**Table 2:** List of mononuclear antibodies

	BM-MNC	
	Patient 1	Patient 2
Characterisation of depleted bone marrow		
Total CD45^–^ cells (%)	64.9	98.4
Total CD45^+^ cells (WBCs) (%)	35.0	1.7
Viability (%)	97.1	99.7
CHECK CD45^–^/7AAD^–^ cells (SS/FS) (%)	23.4	17.3
Viable CD45^+^/7AAD^–^/CD34^+^ cells (%)	0.55	0.96
Characterisation of progenitor cells among		
Viable CD45^–^/7AAD^–^/CD105^+^ cells (%)	0.8	0.1
Viable CD45^–^/7AAD^–^/CD73^+^ cells (%)	0.5	1.0
Viable CD45^–^/7AAD^–^/CD166^+^ cells (%)	0.1	0.01
Viable CD45^–^/7AAD^–^/CD90^+^ cells (%)	0.5	0.2
Viable CD45^–^/7AAD^–^/CD14^+^ cells (%)	0.4	0.6
Viable CD45^–^/7AAD^–^/CD3^+^ cells (%)	0.03	0.1
Viable CD45^–^/7AAD^–^/CD34^+^ cells (%)	0.1	0.01

*7AAD* 7-aminoactinomycin D, *BM-MNC* bone marrow-derived mononuclear cells, *FS* forward scatter, *SS* side scatter, *WBCs* white blood cells

### Anti-inflammatory and neuroprotective properties of BM-MNC in vitro

**Fig. 2:**
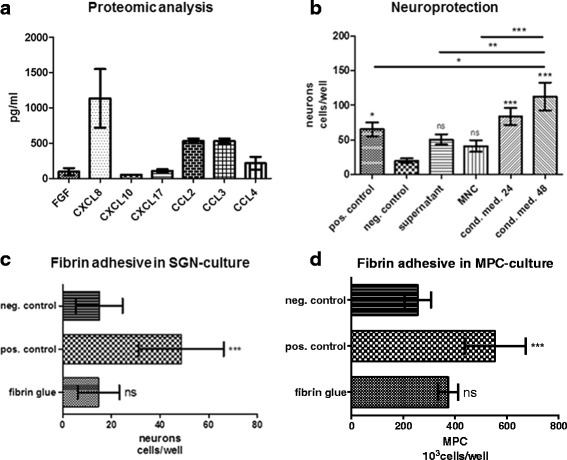
Demonstration of the immunomodulating and neuroprotective secretome of BM-MNC in vitro and generation of biohybrid electrode arrays. **a** Proteomic analysis of bone marrow supernatant. Immediately after centrifugation of the bone marrow, the BM-MNC-containing plasma supernatant was obtained and stored ice-cold until proteomic analysis. Different cytokines, chemokines, and growth factors were present at biologically relevant concentrations in the supernatant. Among these, factors that promote wound healing and modulate and control neuroinflammation were identified. **b** Neuroprotective effect of BM-MNC. Surviving spiral ganglion neurons (*SGN*) were quantified for each treatment condition and compared to the medium (serum-free culture medium; *neg. control*) and to the positive control (medium supplemented with BDNF; *pos. control*). Supernatant was obtained immediately after centrifugation of bone marrow using the RegenKit-THT tubes from each patient. The term BM-MNC denotes the mononuclear cell fraction re-suspended in supernatant. Conditioned medium was obtained after 24 or 48 h (*cond. med. 24/48*) cultivation of the BM-MNC solution in serum-free culture medium. When compared to the negative control, significantly increased survival was determined in the positive control as well as after treatment with conditioned medium. **c**, **d** Biocompatibility of fibrin adhesive tested in well-established in vitro culture assays. Human MPC and SGN isolated from rodents were treated with fibrin adhesive and the survival was compared to the positive control (medium supplemented with BDNF) as well as to cultivation in medium without supplements (*neg. control*). Cell survival was not altered in the presence of fibrin adhesive when compared to the medium control. Values are presented as the mean with standard deviation. *p < 0.1, **p < 0.01, ***p < 0.001. *FGF* fibroblast growth factor, *MNC* mononuclear cells, *MSC* mesenchymal stem cells, *ns* not significant

To characterize the function of the transplanted cells, we assayed a panel of cytokines, chemokines, and angiogenic factors from the transplant material (Fig. 2a). Since demonstration of neuroprotective effects in vivo in humans is currently impossible, a well-established in vitro model derived from neonatal rats was used to study the impact of BM-MNC on the survival of SGN. A neuroprotective effect similar to the one achieved by treatment with BDNF (positive control) was observed by treating the cultures with the cell suspension or with the plasma supernatant obtained from the bone marrow (Fig. 2b). Ex vivo monitoring of the number of neurons showed significantly increased survival when the cultures were treated with conditioned medium harvested after 24 or 48 h (cond. med. 24 or 48) cultivation of BM-MNC. A mean neuronal survival of 112.63 ± 57.14 (mean ± SD) cells per well was observed after the treatment with supernatant collected 48 h after seeding of the BM-MNC. This treatment condition led to the highest survival determined, and nearly doubled the effect achieved by BDNF (Fig. 2b; *p* < 0.001). After treatment with supernatant obtained after 24 h cultivation of BM-MNC, 83.88 ± 34.53 cells per well were counted. Compared to the positive control group (i.e., treatment with BDNF; 65.38 ± 28.40 cells per well), a highly significant increase of survival was obtained (*p* < 0.001). The growth factors, cytokines, and chemokines that were released from BM-MNC (Fig. 2a) could be responsible for the observed neuroprotective effect. We therefore could expect these cells to operate as a neuroprotective and immunomodulatory agent in the vivid inner ear system.

### Biocompatibility of fibrin adhesive

For transplantation of autologous MNC to the inner ear during cochlear implantation, a simple and effective coating procedure was developed. In order to facilitate local factor delivery without migration of the cells off the surface, a fibrin adhesive was added to the cultures of human mesenchymal progenitor cells and isolated spiral ganglion neurons. Neither cell type was negatively influenced by the fibrin adhesive (Fig. 2c and d).

### Similar insertion forces of biohybrid and regular cochlear implant electrodes

**Fig. 3:**
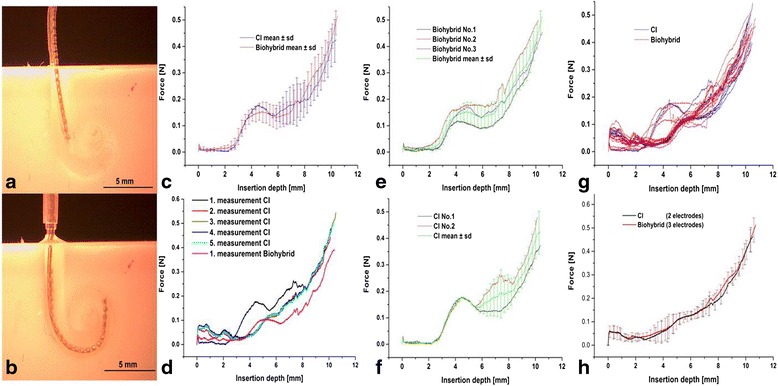
Determination of insertion forces in a cochlear model. **a** Electrode insertion for the measurement of force development due to implantation of a cell-coated electrode compared to uncoated electrode. Human-adjusted cochlear models were used. Electrodes were pre-inserted for 7 mm prior to the start of the measurements. This depth denoting the actual start of the registration of the insertion forces was defined as 0 mm. **b** At the maximum insertion depth of 12 mm, the electrode is regularly jollied. A total of five electrodes (two non-coated (*CI*) and three coated (*biohybrid*) cochlear implant electrodes) were used for the measurement of insertion forces. Each electrode was inserted repeatedly five times. **c** The insertion force was measured from 0 to the 12-mm insertion depth. Here, the mean forces measured at first insertion of each electrode without coating (*blue*) and with coating (*red*) are shown. There was no difference in the insertion forces between the coated and the non-coated electrodes. **d** One electrode was analysed for its stiffness compared to the coating, performing five measures without coating. Then, after coating of the same electrode with the fibrin cell solution, another measurement of insertion forces was performed showing that there are no increases in insertion forces due to the fibrin coating. **e** The first insertion of each biohybrid electrode (biohybrid) is depicted here (No.1–3) as well as the mean and standard deviation of all first biohybrid insertion forces. **f** The first insertion of each regular cochlear implant electrode (CI) is depicted here (No.1 and 2) as well as the mean and standard deviation of all first insertion forces. None of the electrodes showed increased insertion forces. **g** Each electrode is depicted here for direct comparison of the behaviour of uncoated and coated electrode. **h** The mean forces (including standard deviation) of each repeated insertion of biohybrid, as well as CI, electrodes show that there is no difference between the coated and the uncoated electrodes

Coating of the electrodes with a cell-containing fibrin layer leads to a discrete thickening of the radius of the electrode. To rule out increased insertion forces and therefore an increase in structural trauma due to the coating, insertion forces were determined utilizing an artificial cochlear model. The insertion forces and, therefore the mechanical properties of the implant, were not altered due to the coating, as was demonstrated by repeated measures (Fig. 3). Thus, insertion trauma due to the coating could be precluded.

### Behaviour of cells under electrode coating

**Fig. 4:**
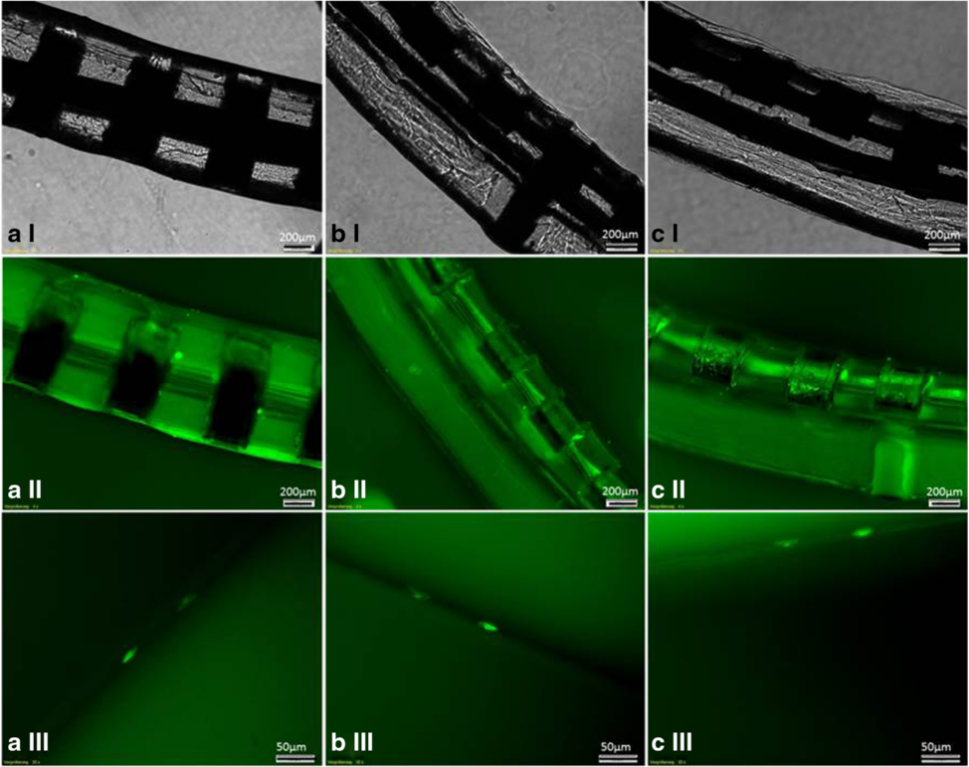
Live-staining of biohybrid electrodes. Light microscopy of the electrodes (*I*) depicting the fibrin layer. Live staining allowed the visualisation of single cells entrapped in the fibrin layer (*II* and *III*). Cells survived on the surface of the electrode without any migration as shown in the micrographs of the same area of the electrode array 3 (**a**), 7 (**b**), and 10 (**c**) days after coating

Fibrin adhesive mixed with BM-MNC built a thin cell-containing layer on the cochlear implant electrode (Fig. 4) allowing proper insertion (Fig. 3).

The biocompatibility of commercially available fibrin adhesive was not only demonstrated in cell culture assays (Fig. 2c and d), but also by determining the survival of encapsulated BM-MNC on the surface of the electrode in vitro using live staining (Fig. 4). Cells survived up to 3 weeks within the fibrin layer in vitro (Fig. 4) and died thereafter. Based on these data, in vivo survival is estimated to be less than 4 weeks.

### Human studies

**Fig. 5:**
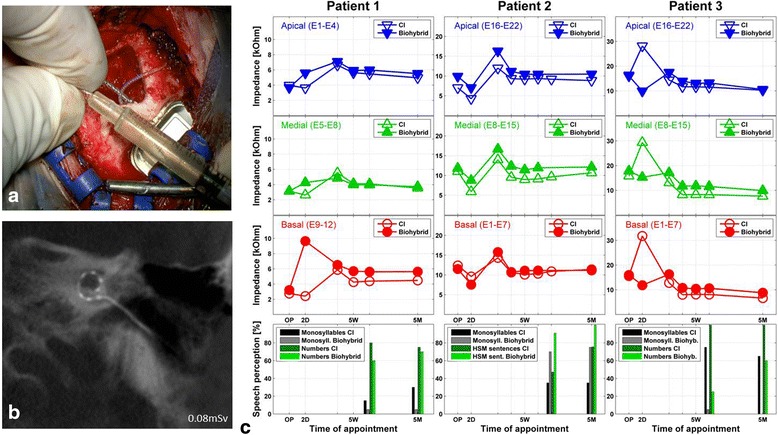
Generation of a biohybrid electrode for human implantation and comparison of performance with standard (*CI*) and cell-coated electrodes (*biohybrid*). **a** Dipping procedure for the intraoperative coating of the electrode with a fibrin cell layer prior to implantation. The BM-MNC solution was mixed with the fibrinogen solution and used for the dip-coating of the electrode. A second dipping into the thrombin solution allowed the stabilization of the coating by conversion of the fibrinogen to fibrin. **b** Cone-beam computed tomography was utilized to check the electrode position immediately after insertion in the operating room. The micrograph shows the correct intracochlear position of the electrode array without any displacement. **c** (*Left panels*) Impedances and speech perception (monosyllables and numbers) of patient one. The patient received a MedEl Synchrony Standard electrode and showed an overall good performance; slightly impaired results on the side with long-term deafness that was treated with the biohybrid electrode are evident. (*Middle panels*) Impedances and speech perception (monosyllables and HSM sentence test) of patient two. The patient received a Cochlear Nucleus CI512 Profile. The results compared favourably to the contralateral side. (*Right panels*) Impedances and speech perception (monosyllables and numbers) of patient three. The patient received a Cochlear Nucleus CI512 Profile electrode. His results with the biohybrid electrode exceeded expectations, taking into consideration the presence of peri/prelingual idiopathic deafness on the side that was treated with the biohybrid electrode. *OP* operation, *2D* initial test (only impedances) on the second day after surgery, *5 W* first fitting week performed 5 weeks after operation, *5 M* control testing 5 months after operation, respectively about three months after the first fitting

Based on previous reports on the safety and efficacy of BN-MNC in other human diseases [36, 37], we set out to test the safety of the cells in conjunction with cochlear implantation. The protocol used for the isolation of BM-MNC and for the generation of a biohybrid electrode was demonstrated to the local authorities and received successful approval for general clinical use without any restrictions. In our human studies, the isolated BM-MNC were added as described above to the fibrin solution and were used to coat the cochlear implant electrode immediately before insertion into the inner ear (Fig. 5a). Patients with profound hearing loss who were candidates for bilateral cochlear implantation were selected for the initial studies. Electrode position was verified using cone beam computed tomography, showing no signs of tip fold-over or any other signs of malpositioning of the electrode (Fig. 5b). The biohybrid electrode performance was compared to the standard non-coated cochlear implant used in the other ear. The electrode impedances and the speech perception were compared between the two implanted ears. All three patients had developed satisfactory speech perception and showed similar impedances on both sides (Fig. 5c). In one of the patients, the speech perception with the biohybrid implant exceeded the performance on the other ear (patient 2), in one it was similar (patient 1), and in one the standard implant outperformed the biohybrid implant (patient 3). None of the subjects demonstrated any adverse effects 5 months after implantation.

## Discussion

In this study, a protocol for the generation of clinically feasible biohybrid electrodes utilizing autologous mononuclear cells (MNC) isolated from bone marrow was developed and tested in vitro. Prior safety studies using MNC in clinical applications have demonstrated the general clinical feasibility of these cells. Thus, the biohybrid electrode has been advanced towards clinical application. Here, for the first time, we provide safety data for the implantation of biohybrid electrodes in cochlear implant patients presenting with challenging conditions prone to poor results.

It has been shown that MNC are involved in the repair of the central nervous system [38–41]. They can be derived from peripheral blood or from the bone marrow, and consist of different cell types including hematopoietic and mesenchymal progenitor cells. The unique capacity of progenitor cells to exert different therapeutic actions depending on the context in which they are transplanted has been reviewed recently [17, 42]. Intrinsic properties of progenitor cells allow for the modulation of the immune system and enable complete regeneration without scar formation after damage or loss of tissue in some species or organs [24, 43]. The decision whether healing will occur with scar tissue formation or whether it may result in scarless tissue healing with functional preservation strongly depends on the quality of the immune reaction [44]. Control of inflammation seems crucial for hearing preservation and for the prevention of fibrotic tissue formation [25] in cochlear implant patients. Thus, immunomodulatory approaches are an attractive therapeutic option [45]. Bone marrow seems to be the most reasonable and natural source of MNC, containing a high percentage of progenitor cells. After depletion of the red blood cells, MNC can be isolated from the bone marrow. Bone marrow-derived MNC (BM-MNC) are known to be neuroprotective [40, 46] and they are able to modulate immune response in favour of the induction of healing and regenerative processes [20, 46, 47]. Utilizing BM-MNC, molecular plasticity (i.e., trophic support and immunomodulation) could potentially be mediated in the inner ear during cochlear implantation. These cells are known for the release of a diversity of cytokines and growth factors [32, 48] and we have analysed a defined panel of cytokines involved in angiogenesis. The cytokines CCL3 and CCL4 modulate neuroinflammation [49, 50]. They also induce the expression of CXCL6 (IL-6). As a mediator for the balance in pro- and anti-inflammatory processes, CXCL6 is known to be neuroprotective [50] and could help restore the physiological environment of the cochlea after damage. Moreover, CXCL8 (IL-8), vascular endothelial growth factor (VEGF) and fibroblast growth factor (FGF)—all known to induce angiogenesis [51, 52]—were produced by MNC isolated from the bone marrow and may be able to increase trophic support in the injured cochlea [53]. FGF is involved in the step-wise differentiation of inner ear sensory epithelia from embryonic stem cells in the mouse [54]. Furthermore, focal delivery of FGF may be able to restore synaptic connections in the inner ear and to promote neuronal outgrowth from spiral ganglion neurons [55]. In addition, it controls the number of progenitor cells [56]. Chemokines such as CCL-2 (MCP-1), CCL3 (MIP-1a), and CCL4 (MIP-1b) were also produced by the isolated cells (Fig. 2a). These factors are known to be involved in neuronal migration, mediation of neuroinflammation, cell proliferation, and in synaptic activity [49, 50, 57] as well as in the chemotaxis and differentiation of neuronal progenitor cells [57]. After noise-induced damage, synaptic connections in the inner ear can be restored [58]. Chemokines and growth factors delivered by BM-MNC may present one approach for synaptic restoration. In addition, such factors could be used to induce or control differentiation processes of local stem cells [59, 60].

Mesenchymal progenitor cells secrete—either constitutively or upon activation [61–65]—large amounts of micro- and nanovesicles, which may contribute to neuroprotection and anti-inflammation. These vesicles are filled with miRNA, proteins, and surface markers that might differ from the ones found in the cell. In an experimental setting, they were able to protect against renal injury in a murine remnant kidney model, supporting renal repair and inhibiting apoptosis [62]. In addition, they have been shown to exert neuroprotection [61, 66]. In this study, the neuroprotective effect of BM-MNC on spiral ganglion neurons was demonstrated in a well-established in vitro model (Fig. 1). However, which cells add to the neuroprotective effect observed in vitro or whether micro- or nanovesicles released from mesenchymal progenitors are responsible for the benefits needs further clarification. A sophisticated in vivo model for electrode insertion trauma has been presented recently [15]. Future studies based on this model are necessary in order to closer investigate the neuroprotective effects and to demonstrate a potential benefit of BM-MNC transplantation for the prevention of insertion trauma in vivo.

Fibrin has frequently been used as a scaffold for tissue engineering, enabling the supply of nutrients and therefore the survival of encapsulated cells [67]. With its excellent biocompatibility [68], it realises a cost-effective and easy to handle approach as a matrix for cell coating. The addition of a fibrin layer on the electrode surface did not alter the insertion behaviour as has been demonstrated in insertional studies (Fig. 3).

Mononuclear cells derived from bone marrow or peripheral blood have been used without any adverse effects in humans for various purposes [26, 32, 36, 37]. Thus, the safety of transplantation of BM-MNC has been demonstrated over the past several years even in closed fluid-filled spaces such as the joints without any adverse effects [36]. In addition, BM-MNC have been successfully implanted recently for the treatment of patients with cerebral palsy [32]. A plethora of in vivo and in vitro studies has concentrated on the use of mesenchymal progenitor or embryonic stem cells [18, 19, 69–71], but only a very few have considered hematopoietic progenitor cells for cell-based therapies for the inner ear [22, 72]. All studies confirm that there is no induction of fibrosis or ossification of the cochlea after local transplantation of progenitor cells. To date, no clinical cell-based applications that target the human inner ear have been developed. We here provide initial safety data after autologous transplantation of BM-MNC via a biohybrid electrode. The short processing time for isolation of BM-MNC from the bone marrow did not induce any delay in surgery. The coating procedure was simple and was performed by the surgeon immediately prior to insertion. Insertion was uncomplicated as was expected from the insertional studies in a human cochlear model. Utilizing this procedure for the isolation of bone marrow-derived progenitor cells could minimize potential epigenetic effects that may occur as a result of plastic adhesion and in vitro expansion. Initial safety requirements have been met in all cases presented here after 6 months, demonstrating a lack of complications and the clinical feasibility of the approach. Further investigations will concentrate on the enhancement of the effect of BM-MNC.

## Conclusion

The herein presented procedure utilizing autologous cells for local factor delivery via biohybrid electrodes in profoundly deaf patients is an initial step towards cell-based regenerative therapies for hearing disorders. Various innovative efforts based on progenitor cells, gene, or molecular therapy for the inner ear have been developed recently [73–75] and could be combined with our procedure for the development of clinically relevant future therapies.

## Abbreviations

7-AAD: 7-Aminoactinomycin D
BDNF: Brain-derived neurotrophic factor
BM-MNC: Bone marrow-derived mononuclear cells
CFSE: 5(6)-Carboxyfluorescein N-hydroxysuccinimidylester
FCS: Fetal calf serum
FGF: Fibroblast growth factor
HBSS: Hank’s balanced buffered solution
mAB: Monoclonal antibody
MNC: Mononuclear cell
MPC: Mesenchymal progenitor cells
PBS: Phosphate-buffered saline
SGC: Spiral ganglion cells
SGN: Spiral ganglion neurons
VEGF: Vascular endothelial growth factor
